# Hypothermia as an Adjunct Therapy to Vesicant-induced Skin Injury

**Published:** 2008-04-30

**Authors:** Thomas W Sawyer, Peggy Nelson

**Affiliations:** Defence Research and Development Canada–Suffield, Alberta, Canada

## Abstract

**Objective:** The notion that cooling vesicant-exposed tissue may ameliorate or prevent resultant injury is not a novel concept. During both World Wars, studies were conducted that investigated this potential mode of therapy with sulfur mustard and seemed to conclude that there might be merit in pursuing this research direction. However, it does not appear that these studies were followed up vigorously, and the literature that describes this work is not readily accessible. In this report, we compare the toxicities of lewisite and sulfur mustard in vitro and in vivo and also provide an overview of historical and recent work on the effect of temperature on the toxicity of these vesicating chemical warfare agents.**Methods:** Tissue culture and animal studies were utilized to examine the effects of hypothermia on vesicant-induced toxicity. **Results:** Cytotoxicity was either significantly delayed (lewisite) or prevented (sulfur mustard) when cultures were maintained at 25°C. However, the effects of hypothermia on sulfur mustard–induced cell death were reversible when the cells were returned to 37°C. Despite these in vitro results, animal studies demonstrated that the therapeutic cooling of both mustard sulfur–exposed and lewisite-exposed skin resulted in dramatic and permanent protection against injury. Cooling also increased the therapeutic window in which drugs were effective against vesicant agents in tissue culture and lewisite-induced skin injury. **Conclusions:** The simple and noninvasive application of cooling measures may not only provide significant therapeutic relief to vesicant-exposed skin but also increase the therapeutic window in which medical countermeasures against vesicant agents are useful.

The chemical warfare (CW) agents lewisite (dichloro [2-chlorovinyl] arsine) and sulfur mustard (bis[(2-chloroethyl]sulfide) have been weaponized and stockpiled in a number of countries around the world.^1^ Although the military use of both compounds is primarily as incapacitants because of their potent vesicant activities, they can also induce a range of toxic pathologies that result in death if exposure is high enough. In historical reports that recount experimental work with these CW agents, it is unusual for the synthetic process by which the munitions-grade products were produced (resulting in varying compositions of the test agents) to be identified. In addition, these records seldom state whether the material used was distilled to produce a higher-purity test article. Therefore, for the purposes of this overview, when it is not possible to identify what the makeup of the actual test agents is, we will refer to them as either sulfur mustard or lewisite, and the distilled products as “HD” and “L,” respectively.

Since its first use in 1917 by the Germans at Ypres, Belgium, sulfur mustard has been utilized in a number of military conflicts. It has well-documented cytotoxic,^2^ mutagenic,^3^^,^^4^ and vesicant properties.^5^^,^^6^ At very high exposures, sulfur mustard also induces profound systemic effects,^7^^–^9 which include the classic signs of a strong alkylating poison as well as a shock-like syndrome that does not respond to treatment.^10^^,^^11^ Other than its well-understood antimitotic effects, the mechanism of toxic action of this CW agent is unknown, and antidotes do not exist against its action.^1^^,^^2^^,^^12^^,^^13^

Lewisite was the result of American efforts to develop an arsenical vesicant to counter German gas use during World War I. After an extensive research program, it was identified as the lead candidate and weaponized in 1918.^14^ The toxicity and vesicant activity of this compound in comparison with that of HD is unclear from the historical literature, with a variety of reports describing it as having less-to-more vesicant activity.^14^ What is clear, however, is that lewisite is a rapid acting and powerful vesicant and its arsenical makeup renders it a potent cytotoxin and blood poison.^15^ In contrast to sulfur mustard, antidotes against this potent vesicant have been developed. British anti-lewisite^16^ as well as other less toxic, more water-soluble chelators^17^^–^21 have been shown to be effective in preventing lewisite toxicity. Lewisite has not been convincingly documented as being used in the battlefield^1^ and its utility as a CW agent has been debated.^14^ Nevertheless, it has been extensively weaponized as a mixture with sulfur mustard, ostensibly to lower the freezing temperature of the latter compound. Undoubtedly, use of this CW mixture would result in painful and difficult-to-treat tissue injury, which heals slowly.

The modulatory effects of temperature on the toxicity of xenobiotics have long been a topic of investigation. During the early part of the last century, the toxicity of colchicine was shown to be dramatically temperature dependent, with its lethality in frogs increasing several hundred folds with only a 12°C increase in temperature.^22^ Subsequent work showed that a large variety of drug actions were temperature dependent and by 1961, a review article identified more than 300 citations in this area.^23^ Indeed, there is a wealth of information available on how temperature modulates the metabolism, distribution, and excretion of a wide range of currently used pharmaceuticals as well as the toxicity of selected xenobiotics.^23^^–^25 Although this research area does not seem to have been as broadly active in recent years, the effects of both hypothermia and hyperthermia have been the topics of interest with respect to improving the efficacy of some cancer chemotherapies.^26^^–^28 Tissue culture studies have shown the increased toxicity of a number of compounds (notably alkylating agents such as Melphalan) with increased temperature. These studies have led to successful clinical trials, using chemotherapy and hyperthermia. Although the success of some of these trials has shown the utility of this approach, the mechanism(s) by which hyperthermia enhances the toxicity of these drugs is unclear, although a number of hypotheses have been advanced, including enhanced membrane permeability, increased metabolic activation to toxic species, increased rate of hydrolysis, increased blood flow, and increased rate of reaction of the drug with critical macromolecules.^29^^–^31

In this report, we briefly compare the toxicities of lewisite and sulfur mustard both in vitro and in vivo and also provide an overview of historical and recent work on the effect of temperature on the toxicity of these vesicating CW agents.

## METHODS

### Cell culture

Seed cultures of J-774, CHO-K1, and HeLa cell lines were obtained from the American Type Culture Collection (Manassas, Va). J-774 cells are a mouse macrophage–derived cell line, CHO-K1 cells are a strain of the epithelial cell line derived from Chinese hamster ovary cells, and HeLa cells were originally cultured from a human cervical carcinoma. The cultures were grown in 10% foetal calf serum in Dulbecco's Modified Eagle Medium supplemented with streptomycin (100 µg/mL) and penicillin (100 IU/mL), and the medium was changed as required. Stock cultures were closely monitored and not allowed to grow to confluency before being subcultured. Test cultures were used just before, or at, confluency. The methods for culturing human skin keratinocytes and chick embryo neurons have previously been described.^32^^–^35

### Chemical treatment

On the day of chemical treatment, the cultures were treated with the CW agent dissolved in ethanol so that the desired final agent concentration was reached at 0.25% ethanol (vol/vol). To assess cytotoxicity, alamarBlue was added (10%, vol/vol) to the cultures and allowed to incubate for the last 3 to 5 hours of the routine 24 hour (cell lines, neurons) or 48-hour (keratinocytes) treatment. The absorbances (570–600 nm) were then read on a Thermomax titerplate reader (Molecular Devices, Sunnyvale, Calif). This assay measures metabolic viability and we have found it to be a reliable indicator of cytotoxicity, yielding results similar to that obtained by more commonly used methods such as the neutral red and MTT assays.^34^ Median lethal concentration values were determined graphically from experiments, utilizing 6 wells per data point. In all cases, values represent data from at least 3 separate experiments. Studies investigating the effects of temperature on HD or L toxicity in neurons and keratinocytes were carried out in humidified CO_2_ incubators set at 25°C, 31°C, or 37°C. Munitions-grade sulfur mustard was distilled at Defence Research and Development Canada–Suffield to greater than 98% purity. Munitions-grade lewisite was distilled and the resultant product consisted of 79.9% dichloro (2-chlorovinyl) arsine (the desired lewisite product), 0.9% bis(2-chlorovinyl) chloroarsine, and 17.9% unknown.^33^

### Animal studies

Male hairless guinea pigs (strain Crl: IAF(HA)-hrBR) were acquired from Charles River Laboratories (St Constant, Quebec, Canada). The animals were acclimated for at least 1 week before experimental use. In conducting this research, the authors adhered to the *Guide to the Care and Use of Experimental Animals* and *The Ethics of Animal Experimentation* published by the Canadian Council on Animal Care. The methodology for HD and L skin exposure, as well as cooling agent-exposed sites, has previously been described.^32^^–^35

## RESULTS AND DISCUSSION

### Historical vesicant hypothermia research

Some of the first work to investigate the effect of lowered temperature on sulfur mustard toxicity appears to have been motivated by a then-popular theory of mustard toxicity. The “hydrochloric acid theory of mustard vesication” (reviewed in Papirmeister et al^2^ hypothesized that the chemical hydrolysis of sulfur mustard within the cell resulted in sufficient liberation of hydrochloric acid so as to cause cytotoxicity. In a review article by Lynch et al,^8^ the authors reasoned that since the rate of sulfur mustard hydrolysis would be much decreased by reducing the temperature, this should provide relief from its toxicity. In these studies, catfish were exposed to a “quarter-saturated” solution of sulfur mustard in tap water for 5 minutes, or goldfish to a “half-saturated” solution of sulfur mustard in tap water for 10 minutes, and then transferred to either room temperature water (24°C–26°C) or cold water (8°C–10°C). Of the 10 catfish that were placed in room temperature water, all died within 28 hours, exhibiting hemorrhages on the fins, tails, and ventral surfaces within 20 hours. Five of 7 catfish placed back into the cold water survived for the 5-day experimental treatment with no signs of skin injury being observed. In the goldfish experiments, all 8 fish returned to room temperature water died within 6 days, while all 7 fish returned to the cooler water survived the 17-day test period. No skin injury was observed in the goldfish experiments. The authors concluded, after conducting further studies demonstrating that the toxicity of hydrochloric acid was not temperature dependent in these 2 species, that “these experiments on fish tend to substantiate the theory of intracellular liberation of acid.”^8^ Although the protective effects of hypothermia against sulfur mustard were demonstrated in these studies, it appears that these experiments largely summarized the work carried out with hypothermia and mustard sulfur to this point in time, since this report was reproduced in its entirety in a history of American medical countermeasures research against gas warfare published in 1926,^36^ with no mention of any additional related work.

Between the World Wars, a great deal of CW research was carried out by a number of countries, since it was anticipated that the next major war would rely heavily on chemical weaponry. However, to our knowledge, there is no record of work carried out on the effect of lowered temperature on sulfur mustard injury. During World War II, while there was a wealth of research that supported the assertion that sulfur mustard–induced skin injury was definitively more severe in hot and humid conditions (either occluded by clothes or in tropical climates),^2^^,^^37^ relatively little work appears to have been carried out on the effects of hypothermia. The focus of much of the work that did occur, appeared to use lowered temperature to decrease the amount of “fixed” (or nonextractable) sulfur mustard in the skin, an endpoint that was believed to be correlated with the severity of skin injury.^37^ The original reporting of this work has not been possible for the authors to obtain. However, intriguing hints exist that point to the efficacy (or lack thereof) of cooling sulfur mustard–exposed tissue. Sir Frederick Banting, codiscoverer of insulin and a major supporter of Canada's chemical biological effort during the World War II, was said to have tested the effects of ice-pack treatment on himself in 1940. He was described as undergoing a 5-minute exposure of 100 mg of sulfur mustard spread over a 6.5 × 1.5-in area on his lower right leg, before blotting the residual liquid off and applying ice. No toxicity was apparent until he had to fight a grass fire the next afternoon and the ice melted, resulting in extremely serious and painful injury. Despite this, the Canadian Medical Research Council apparently considered the ice treatment to be a success.^38^

**Figure 1:**
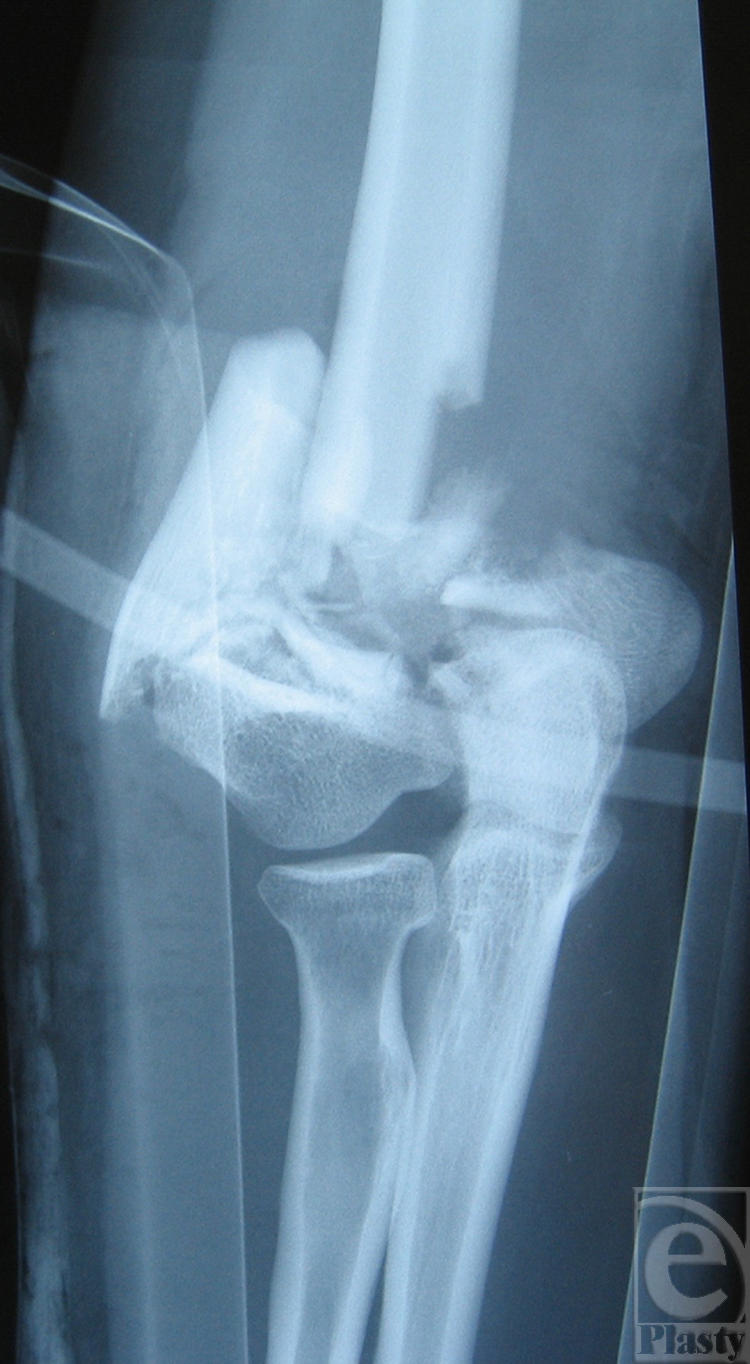
Reproduction of a page from a 1943 Chemical Warfare Laboratories (Ottawa, Ontario, Canada) physiological note referring to successful therapeutic ice-pack treatments to sulfur mustard–skin injury. Sulfur mustard is referred to as “H” while “S” refers to nitrogen mustard-2.

Additional intriguing evidence that ice-pack treatment of sulfur mustard–exposed skin might be of utility is a record of a meeting held by the Physiological Mechanisms Committee in New York City.^39^ On the last page of this document (Fig 1), there is a short discussion on the efficacy of ice-pack treatment on sulfur mustard–exposed skin with respect to lowering the amount of nonextractable sulfur mustard, and preventing skin injury. Three years later, a review of what appears to be related work is included in a treatise published, describing the Allied CW agent research effort.^40^ The chapter on mechanisms of sulphur mustard-induced skin injury^37^ details considerable effort demonstrating the relationship between the amount of nonextractable sulfur mustard in the skin and resultant injury. It documented that in human skin, in contrast to pig and rabbit skin, sulfur mustard is almost maximally fixed within 2 minutes of exposure. The ice-pack studies subsequently described were viewed as confirmatory evidence that there was virtually no significant reservoir of unreacted sulfur mustard in human skin very shortly after exposure. In this work, humans and pigs were exposed to liquid mustard for 10 minutes, decontaminated with petroleum ether, and then ice-packs were applied to the exposure sites. The results of these experiments were described as “In man (Table 8), the ice-pack treatment did not significantly affect either the amount of fixed H as determined 24 hours after exposure or the severity of the lesion that developed. In the pig (Table 9), on the other hand, the ice-pack treatment resulted in the fixation of considerably less H than would otherwise have been the case, and in the partial or complete inhibition of visible injury development.”^37^ Thus, it was concluded that in the pig, cooling exposed skin decreased the rate of sulfur mustard fixation, allowing it to be slowly carried away by body fluids during the cooling treatment, whereas in man, cooling was ineffective because virtually all sulfur mustard fixation occurred during exposure. Interestingly, although Renshaw chose not to include cooling as a possible treatment modality in his summary to this chapter, he referred to “Canadian experiments” as demonstrating that if thorough decontamination of sulfur mustard–exposed skin is not carried out then ice-pack treatment does reduce the severity of the skin injury. To this point, we have not been able to locate the original documentation of these studies.

### Effect of hypothermia on vesicant-induced toxicity

Our interest in the potential protective effects of cooling HD-exposed tissue did not arise from historical considerations or from the hypothesis that hypothermia would prevent or ameliorate the initial lesions that HD causes to eventually cause toxicity. Rather, our motivation for examining temperature as a potential modulator of mustard toxicity was a result of work carried out with a class of drugs that conferred significant protection against HD toxicity in vitro. These compounds were nitric oxide synthase inhibitors with structural similarities to D/L-nitroarginine methyl ester D/L-NAME). Although they were shown to confer up to 300% protection against HD (in terms of median lethal concentration), this protection was *not* mediated through nitric oxide synthase inhibition.^41^^–^43 While these findings were in themselves interesting, what was also intriguing was that the efficacy of L-NAME in cultures treated *after* HD exposure, remained maximal at 3 hours post–HD treatment, and then only slowly declined over the next 5 hours. These findings were consistent with HD rapidly initiating its effects and setting into motion metabolic events that resulted in a lesion (inhibited by L-NAME) being first expressed by approximately 3 hours post–HD treatment and fully expressed by about 8 hours post–HD treatment. This hypothesis could be easily tested by altering the incubation temperatures of HD-treated cultures and examining the effect of temperature on the efficacy of L-NAME posttreatment on HD toxicity, as a function of time. These studies were subsequently carried out. Similar studies were also carried out with L and the chelator dimercaptosuccinic acid (DMSA). Although both HD and L are vesicant in nature, they are very different in how they exert their respective toxicities. A comparison of their toxic effects was thus carried out both in vitro and in vivo.

**Figure 2:**
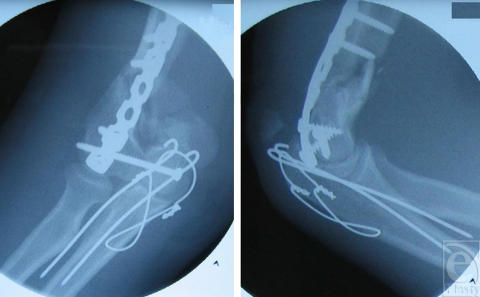
Effect of vesicant vapor exposure on hairless guinea pig skin. Vapor exposures of either distilled sulfur mustard (HD) or distilled lewisite (L) were carried out on hairless guinea pig skin for 2, 4, or 6 minutes. Results show the development of skin lesions at 7, 24, and 72 hours. Results depict the appearance of lesions on 3 animals and are representative of several different experiments.

The toxicity of the 2 agents was initially compared in cell cultures of widely varying origins (Table 1). The toxicity of L was at least 2 orders of magnitude greater than that of HD in differentiated chick embryo neurons, proliferating cultures of first passage human skin keratinocytes, CHO-K1, and HeLa cells, and 70 times more toxic in J-774 cells. The toxicities of HD and L were also assessed in studies using hairless guinea pigs (Fig 2), an animal model that has been shown to develop microblistering of the epidermis when the skin is exposed to HD vapor for 2 to 8 minutes.^44^ In our laboratory, these 2 agents produced skin injury in this animal model in different fashions.^32^^,^^33^ Four- and six-minute HD exposures slowly produced visible lesions that were maximal by 72 hours. In contrast, 2- to 6-minute L vapor exposures produced lesions of much greater severity. Lewisite-induced skin injury progressed very rapidly so that blood scabs were well formed at all exposure sites by 24 hours and of maximal severity by approximately 48 hours. Although the vapor pressure of L is several times higher than that of HD, it is significantly more cytotoxic and produces much more severe skin injury than HD does under similar exposure conditions.

### Effect of hypothermia on sulfur mustard–induced toxicity

**Figure 3:**
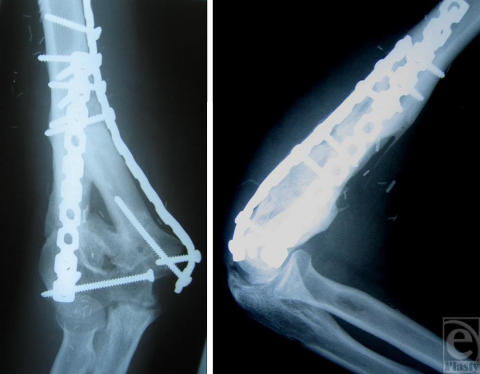
Effect of incubation temperature on the development of distilled sulfur mustard (HD) and distilled lewisite (L) toxicity in human skin keratinocytes. Proliferating cultures of first passage neonatal human skin keratinocytes were treated with either HD (A) or L (B), and then incubated at 25°C, 31°C, or 37°C. At 1, 2, 3, or 4 days after treatment, unique test cultures were assayed for viability using alamarBlue. The HD-treated cultures assayed at 4 days, were refed, and then incubated for an additional 24 hours at 37°C before viability assessment. Data represent the mean ± SEM of three different experiments using tissue from three different donors, and was analyzed by analysis of variance and post hoc Dunnett's Method Comparison testing. *Significantly different from the median lethal concentrationobtained at 24 hours at 37°C, *P* < .05.

The effect of temperature on the toxicity of HD has been well characterized in first passage human skin keratinocyte cultures of varying differentiative states.^35^ The toxicity of HD gradually declined as the cells were tested in proliferating, just-confluent, or post–confluent stages of culture. Nevertheless, in all cases, HD toxicity was significantly reduced by holding the cultures at 25°C, as opposed to 37°C. The effects of temperature were further examined in proliferating cultures treated with HD^32^ and held at 25°C, 31°C, or 37°C. At 37°C, HD toxicity was rapidly expressed and maximal by 48 hours postexposure. When the cells were incubated at 31°C, the 24-hour toxicity was significantly less than that exhibited at 37°C, but the cells then progressively lost viability over the ensuing 4 days. At 25°C, HD toxicity on day 1 was similar to that at 31°C, but increased only marginally with time so that by day 4, toxicity was still significantly less than that exhibited in cultures incubated at 31°C or 37°C (Fig 3A). Extensive confirmatory vidence of the protective effect of lowered temperature against HD toxicity was also obtained when apoptotic endpoints (morphology, DNA fragmentation) were utilized.^32^ The protective effects of lowered temperature were not persistent. When cultures incubated at the lowered temperature for 4 days were switched back to 37°C, the toxicity after 24 hours was similar to those of cultures incubated at 37°C for the entire 5-day time period (Fig 3A).

**Figure 4:**
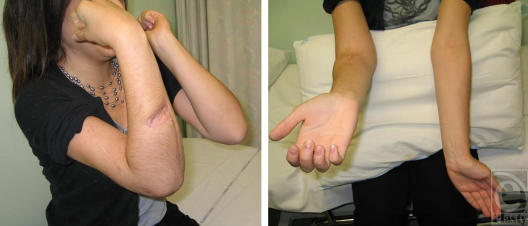
Effect of cooling on skin lesions induced by sulfur mustard (HD) and lewisite (L) vapor in hairless guinea pigs. Animals were exposed to HD or L vapor for 2, 4, and 6 minutes. The exposure sites were then left at either room temperature (top panels) or cooled (approximately 10°C) for 4 hours (L) or 6 hours (HD, bottom panels). The figure depicts the appearance of skin lesions on three animals at 72 hours posttreatment.

Although the transient protective effects of cooling HD-exposed cultures were not promising with respect to obtaining positive outcomes in animal work, cooling of HD-exposed hairless guinea pig skin at 10°C for 6 hours (Fig 4) or at 5°C for 4 hours was protective in comparison with animals whose exposure sites were left at room temperature whereas similar cooling regimens at 15°C were not. Decreased, but still significant protection was also obtained if cooling (5°C) was delayed by 1 hour before 5 hours of cooling.^32^

Similar, but less extensive work examining the effect of cooling HD-exposed skin was also carried out using 20-kg domestic swine.^45^ In these studies, longer HD vapor exposure times (4–16 minutes) of the abdominal area were necessary to produce skin injury. The 4-hour ice-pack treatments used to obtain maximal protective effects in this animal model only required the skin to be cooled to approximately 15°C to 20°C. Cooling of HD-exposed skin consistently resulted in less injury than exposure sites left at room temperature. However, the severity of the lesions produced by HD exposure, as well as the protection afforded by cooling, appeared to be significantly more variable in swine than that observed in hairless guinea pigs. As the structure of swine skin is significantly closer to that of human skin than is guinea pig skin, this may suggest a similar variable response in humans.

**Figure 5:**
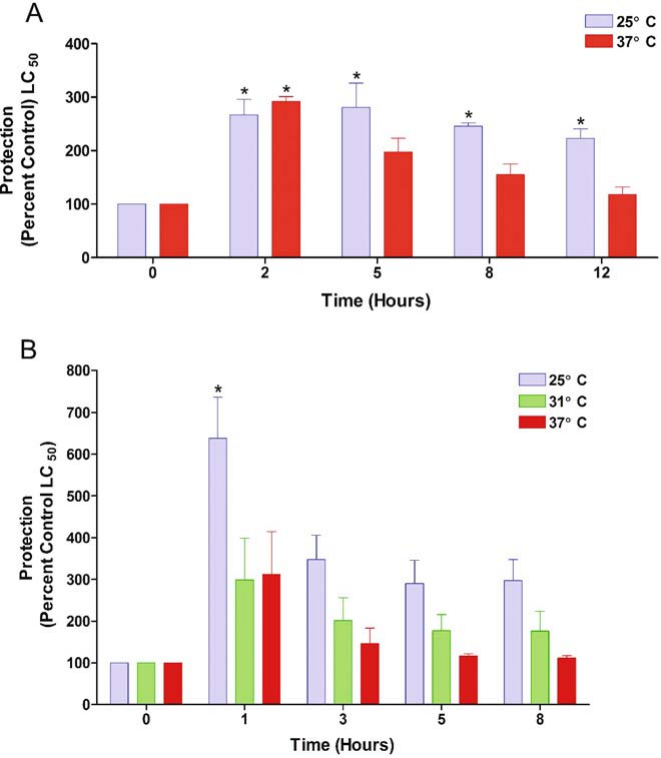
Effect of incubation temperature on the efficacy of protective drug treatments in cell cultures exposed to sulfur mustard (A) or lewisite (B). Mature chick embryo neuron cultures were treated with 5.0 mM L-nitroarginine methyl ester (L-NAME) at varying time intervals after HD exposure of cultures incubated at 25°C or 37°C (A) and proliferating cultures of first passage cultures of human skin keratinocytes were treated with 1.0 mM demercaptosuccinic acid (DMSA) at varying time intervals after L exposure of cultures incubated at 25°C, 31°C, or 37°C (B). Test cultures were assayed for viability using alamarBlue™. Results represent the mean ±SEM of three separate experiments using tissue from different donors, and were analyzed by analysis of variance and post hoc Tukey HSD Multiple Comparison testing. ⋆ Significantly different from the median lethal concentration of agent-only–treated cultures within the same test temperature, *P* < .05.

As stated previously, our original goal in examining the effect of temperature on vesicant-induced toxicity was to ascertain whether cooling HD-treated cell cultures would result in an increase in the therapeutic window in which L-NAME was protective. This drug is effective against HD in just-confluent cultures of human skin keratinocytes. However, the extremely rapid decline in HD toxicity with culture age, especially as the cultures became confluent and differentiation was initiated, precluded reproducible protection studies in this system. We, therefore, carried out this work in differentiated primary chick embryo neuron cultures, a system that we have found to be very sensitive to the effects of HD, sensitive to the protective effects of L-NAME, and to be predictive of results obtained in human skin keratinocytes.^35^ Lowering the incubation temperature of HD-exposed neurons significantly lengthened the period of time in which L-NAME was still of therapeutic value (Fig 5A). At 37°C, 5.0 mM L-NAME was maximally effective when added 2 hours after the neurons had been exposed to HD. This protection then gradually decreased until no significant advantage was obtained 12 hours post–HD exposure. At 25°C, maximal protection could still be observed when adding the L-NAME up to 12 hours after HD exposure. No efforts were made to duplicate these results in the hairless guinea pig model, since L-NAME was not found to be protective in this model system, probably due to difficulties in obtaining and maintaining sufficiently high concentrations at the HD target sites.^35^

### Effect of hypothermia on lewisite-induced toxicity

In contrast to the results obtained with HD, keratinocyte cultures exposed to L and incubated at 25°C rapidly lost viability with time (Fig 3B). At 37°C, L toxicity was almost maximal by 24 hours and decreased only slightly during the next 3 days. At 24 hours, in cultures incubated at 31°C, the toxicity of L was reduced (but not statistically significant) in comparison with that obtained at 37°C. However, by 48 hours, this protective effect was lost and median lethal concentration values were similar to those obtained at 37°C. The protective effect of lowered temperature was dramatic when the treated cultures were incubated at 25°C. The median lethal concentration values obtained at 24 hours were more than 12 times higher than those at 37°C. However, these protective effects were rapidly lost over the following days. Clearly, the development of HD toxicity in this culture system is more temperature dependent than that of L.

**Figure 6:**
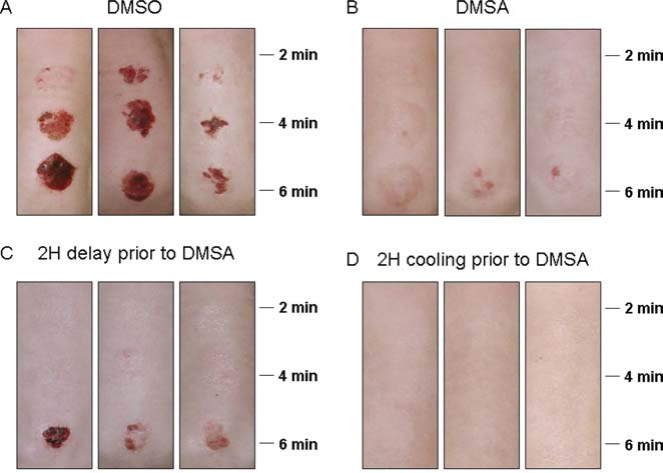
Effect of incubation temperature on the efficacy of protective demercaptosuccinic acid (DMSA) treatment after exposure of distilled lewisite (L) on hairless guinea pig skin. Animals were exposed to L vapor for 2, 4, or 6 minutes. At 2 minutes posttreatment, the exposure sites were treated topically with 50 µL of vehicle (DMSO) only; (A) or DMSA (50 mg/mL, B) for 1 hour. In additional treatment groups, the L exposure sites were left for 2 hours at room temperature before being treated with DMSA (50 µL of 50 mg/mL, C) for 1 hour, or the exposure sites were cooled at approximately 10°C for 2 hour before the 1-hour treatment with DMSA (D). Three animals were used for each treatment and the photographs depict the appearance of the lesions at 3 days posttreatment.

Lewisite produces much more toxicity in a shorter time frame, both in vitro and in vivo than does HD. Thus, the results of the cooling experiments utilizing hairless guinea pigs exposed to L were unexpected.^33^ Not only were the lesions caused by 2 to 6 minutes L vapor exposures very significantly reduced by cooling at 10°C for as little as 30 minutes, but protection could still be obtained by cooling up to 1 hour post–L exposure. Perhaps, most surprising was that the very serious skin injury produced by L vapor exposure could almost be *totally* eliminated by cooling (Fig 4). Protection studies using DMSA and L were carried out in human skin keratinocytes. In contrast to the protective effects of l-NAME against HD toxicity, which are only exhibited in differentiating cultures, DMSA is also protective against L toxicity in proliferating cultures. Cultures were held at 25°C, 31°C, or 37°C exposed to L, and then treated with 1.0 mM DMSA at 1, 3, 5, or 8 hours postexposure. After DMSA treatment, all cultures were returned to a 37°C incubator for the remainder of the 48 hour L exposure period. When the cultures were DMSA-treated at 1 hour post–L exposure, significant protection against L was obtained only at 25°C, although the viability at the other 2 temperatures was elevated. After this, the protective effects of DMSA rapidly declined with time at all temperatures (Fig 5B). The relative success of this in vitro work led us to examine the possibility that hypothermia may also result in an extended DMSA therapeutic window against L in hairless guinea pigs.^33^ In comparison with vehicle [dimethylsulphoxide (DMSO), Fig 6A] treatment only, a 1-hour DMSA treatment immediately after L vapor exposure resulted in very significant protection, with only minor skin injury evident at the 6-minute exposure sites (Fig 6B). Delaying the DMSA treatment by 2 hours resulted in very good protection at the 2-minute and 4-minute L exposure sites, but not at the 6-minute L exposure sites (Fig 6C). The severity of skin injury from this latter treatment was similar to that obtained when the exposure sites were cooled for 2 hours.^33^ Combining these 2 treatments, so that DMSA was applied after 2 hours of cooling, totally eliminated all signs of skin injury (Fig 6D). Even close examination of the exposure sites failed to detect any difference from the surrounding tissue.

## SUMMARY

There is a temptation to dismiss records of historical military research as primitive and/or lacking in detail. Much of this work was driven by real-time military requirements, and results not relevant to the stated objectives of the project were often not recorded or pursued. Thus, the scientific detail required to accurately assess some of the historical defense literature is often lacking. Nevertheless, the scientists involved in these endeavors, particularly those working under the urgency of current or anticipated world conflict, were often the finest that their countries had to offer. These studies also utilized human volunteers when deemed necessary, an option that is not available to modern day CW researchers because of current human ethical guidelines. It is thus important to objectively assess the potential practical importance of findings, using tissue culture and animals in light of historical results, especially those that include human studies.

Tissue culture data have definitively shown that cooling slows the development of both HD- and L-induced cytotoxicity, and support the hypothesis that their toxicity is the result of a metabolic cascade of events. This in vitro protection against HD is reversible in nature and there is historical evidence supporting the notion that sulfur mustard is “fixed” (nonextractable) very quickly in human tissue, and thus not amenable to cooling therapy. From these findings, one might conclude that cooling HD-exposed human skin would not result in any significant permanent protective effects. However, recent work has demonstrated that there is a very significant reservoir of unreacted HD in both human and pig skin for up to 24 hours after contamination of the skin surface in vitro, thus elevating the likelihood that cooling HD-contaminated skin might indeed reduce resultant skin injury.^46^ Lewisite is less likely to be fixed in the skin than would the alkylating agent HD, and it seems probable that cooling L-exposed skin may also be effective in providing protection. As originally postulated by Renshaw,^37^ with sulfur mustard, this would occur by lowering its (bio)chemical reactivity, while allowing the systemic circulation to slowly transport the unreacted agent away from the site of exposure. It is also likely that an important aspect of cooling vesicant-exposed skin would be by increasing the therapeutic window in which medical countermeasures against these CW agents may be applied. Tissue culture studies show that cooling does increase this window of opportunity for protective drug treatments, most probably by slowing metabolism. Therapeutic medical intervention of HD- or L-induced injury may be possible by blocking events downstream from the initial lesion, whether the agent is “fixed” or not. This approach may presently be successful with chelation therapy against L, and in the future with HD when effective medical countermeasures against this agent are developed. The noninvasive and simple act of cooling vesicant-exposed skin, while not practical for large body surfaces, may provide a facile means of reducing vesicant-induced cutaneous injury.

## Figures and Tables

**Table 1:** Comparative toxicity of vesicant agents in tissue culture*

	Median lethal concentration	
	Distilled sulfur mustard	Lewisite
Human skin keratinocytes (proliferating)	4.16 ± 1.29 × 10^−5^	1.88 ± 0.24 × 10^−7^
Chick embryo neurons (differentiated)	5.25 ± 1.62 × 10^−5^	2.99 ± 1.78 × 10^−7^
CHO-K1	2.45 ± 0.37 × 10^−4^	1.83 ± 0.24 × 10^−6^
HeLa	1.93 ± 0.31 × 10^−4^	1.91 ± 0.19 × 10^−6^
J-774	4.53 ± 0.23 × 10^−5^	6.27 ± 0.19 × 10^−7^

*Values represent mean ± SD of at least 3 separate
